# Genetic analysis of the regulation of the voltage-gated calcium channel homolog Cch1 by the γ subunit homolog Ecm7 and cortical ER protein Scs2 in yeast

**DOI:** 10.1371/journal.pone.0181436

**Published:** 2017-07-24

**Authors:** Takafumi Kato, Aya Kubo, Tatsuya Nagayama, Shinichiro Kume, Chikara Tanaka, Yoshitaka Nakayama, Kazuko Iida, Hidetoshi Iida

**Affiliations:** 1 Department of Biology, Tokyo Gakugei University, Nukui kita-machi, Koganei, Tokyo, Japan; 2 Laboratory of Biomembrane, Tokyo Metropolitan Institute of Medical Science Kamikitazawa, Setagaya, Tokyo, Japan; Kindai University, JAPAN

## Abstract

The yeast Cch1/Mid1 Ca^2+^ channel is equivalent to animal voltage-gated Ca^2+^ channels and activated in cells incubated in low Ca^2+^ medium. We herein investigated the third subunit, Ecm7, under the same cell culture conditions. The deletion of *ECM7* slightly lowered Ca^2+^ influx activity in the *CNB1*^+^ background, in which calcineurin potentially dephosphorylates Cch1, but markedly lowered this activity in the *cnb1*Δ background. The deletion of the C-terminal cytoplasmic region of Ecm7 also reduced Ca^2+^ influx activity. We identified a novel Cch1-interacting protein, Scs2, which is known as a cortical endoplasmic reticulum membrane protein. The deletion of *SCS2* did not affect Ca^2+^ influx activity when calcineurin was inhibited by FK506, but enhanced this activity by 35% when the enzyme was not inhibited. However, this enhancement was canceled by the deletion of *ECM7*. These results suggest that Cch1/Mid1 is regulated differentially by Ecm7 and Scs2 in a manner that is dependent on the phosphorylation status of Cch1.

## Introduction

Ca^2+^ is a second messenger that induces changes in many fundamental cellular events, including enzyme activation, secretion, and gene expression [1]. Therefore, the permeation of Ca^2+^ through cellular membranes is crucial and strictly regulated by a number of transporters and channels, one of which belongs to the voltage-gated Ca^2+^ channel (VGCC) superfamily. Animal VGGCs are composed of four subunits, such as the pore-forming α_1_ subunit and three auxiliary subunits, α_2_/δ, β, and γ, and are regulated by membrane-located and cytoplasmic proteins [2, 3]. The role of the auxiliary subunits is to modulate the level of expression and voltage dependence of the α_1_ subunit. The γ subunit generally exerts smaller effects.

**Fig 1:**
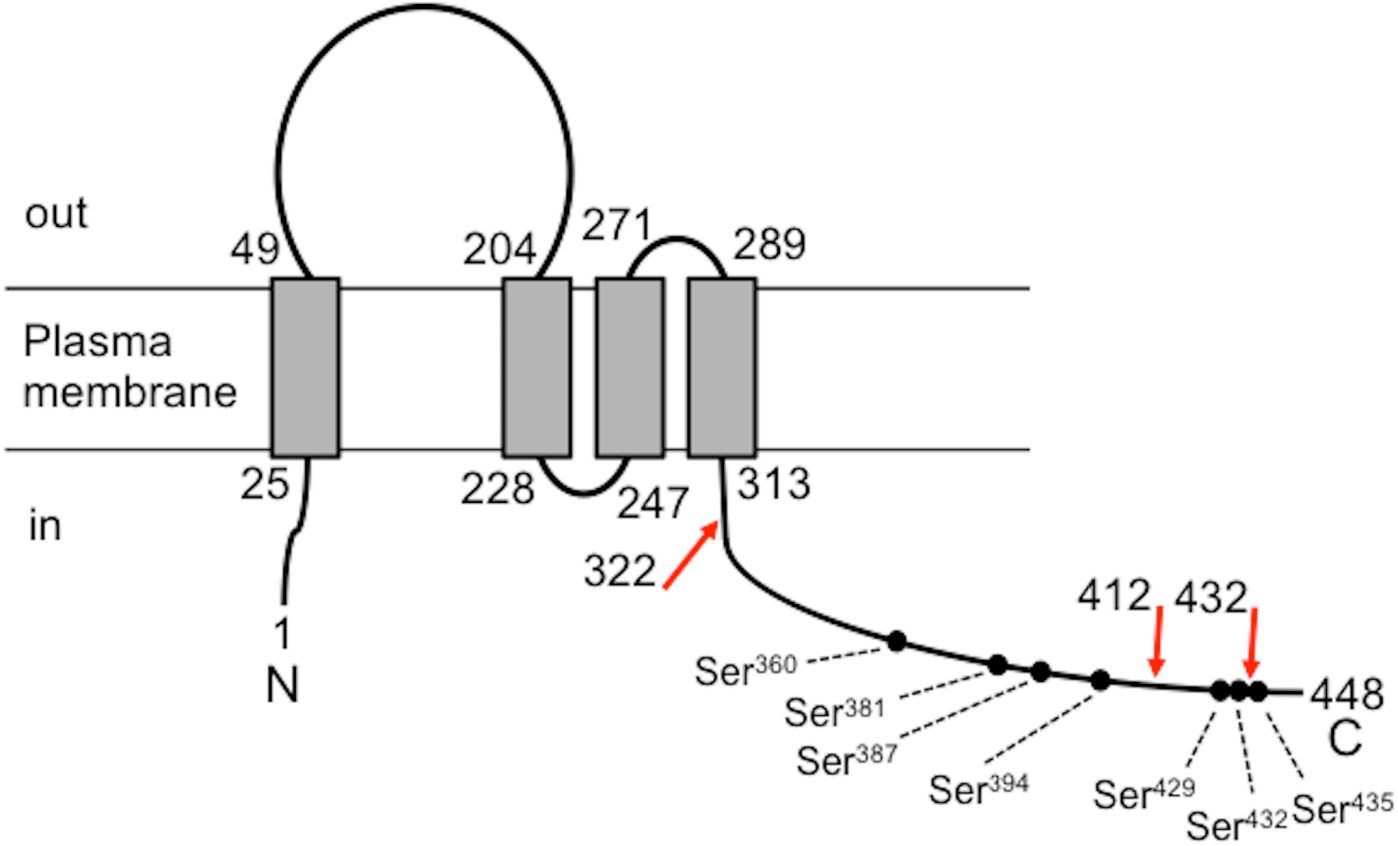
Schematic diagram of Ecm7. This diagram was drawn based on transmembrane prediction with the TMHMM Server v 2.0 (http://www.cbs.dtu.dk/services/TMHMM/). Circles represent phosphorylation sites determined by a mass spectrometry-based proteomic analysis [26]. The red arrows represent cut sites to produce C-terminally truncated forms of Ecm7.

The yeast *Saccharomyces cerevisiae* has a VGCC homolog that is mainly composed of the α_1_ subunit homolog, Cch1, and α_2_/δ-like protein, Mid1 [4–6]. Mid1 is essential for the Ca^2+^ influx activity of Cch1. Both proteins constitute a high affinity Ca^2+^ influx system (HACS) [7], which is often called the Cch1/Mid1 channel and become active in cells grown in synthetic medium with low Ca^2+^ concentration (100 μM). A homolog of the γ subunit is Ecm7 [8]. Ecm7 is a four-transmembrane protein with cytoplasmic N- and C-termini and putative phosphorylation sites (see Fig 1). The role of Ecm7 in Ca^2+^ influx was studied initially by Cunningham and co-workers, but their assay was performed for yeast cells grown in complex YPD medium with high Ca^2+^concentration (~5 mM) [8]. It is known that the Cch1/Mid1 channel is barely active in such cells [7]. Therefore, this circumstance warrants a further study using Cch1/Mid1-active cells grown in synthetic medium with low Ca^2+^ concentration. β subunit homologs are not present in the *S*. *cerevisiae* genome. The Cch1/Mid1 channel is required for Ca^2+^ influx induced by various stimuli, including the mating pheromone α-factor [4–6], endoplasmic reticulum stress [9], hyperosmotic stress [10], hexose re-addition [11] alkaline stress [12], and ethanol stress [13]. The wide responsiveness of the Cch1/Mid1 channel implies the presence of various regulators. The most prominent regulator is calcineurin, a protein phosphatase comprising the catalytic subunit Cna1 or Cna2 and the regulatory subunit Cnb1. When the calcineurin inhibitor, FK506, was added to yeast cells exposed to α-factor or endoplasmic reticulum (ER) stress, Ca^2+^ influx through the Cch1/Mid1 channel was found to markedly increase [7, 9]. In addition, it is shown that Cch1 is phosphorylated *in vivo* in cells treated with either FK506 or the ER-stressor tunicamycin or both and is dephosphorylated by calcineurin *in vitro* [14]. Thus, the phosphorylation status could be important for the activity of this channel.

Scs2 is an integral ER membrane protein homologous to VAP (vesicle-associated membrane protein-associated protein) and connects the ER to the plasma membrane to regulate the level of phosphatidylinositol 4-phosphate (PI4P) in the plasma membrane by controlling the access of another integral ER membrane protein, Sac1 phosphatase, to its substrate PI4P [15, 16]. At the ER/plasma membrane contact sites, interactions between Scs2 and the oxysterol-binding homology protein Osh3 activates ER-localized Sac1. Scs2 also interacts with the septin Shs1 to create the ER diffusion barrier, by which diffusion of integral ER proteins is restricted between the ER in the mother cell and the ER in the daughter cell [17, 18]. Based on a genome-wide screen for mutants with altered Ca^2+^ uptake, Scs2 has been identified as a member of proteins whose defect results in a deficiency in a low affinity Ca^2+^ influx system (LACS) that is stimulated by α-factor in YPD medium [8].

In the present study, we show genetically that Ecm7 and Scs2, which we identified as a new Cch1-binding protein, differentially regulate the Ca^2+^ influx activity of the Cch1/Mid1 channel in a manner that depends potentially on the phosphorylation status of this channel or its regulators.

## Materials and methods

### Yeast strains and plasmids

The *S*. *cerevisiae* strains and plasmids used in this study are listed in Tables 1 and 2, respectively. We used standard methods to manipulate yeast and genetic materials [19, 20]. *Escherichia coli* strain XL10-Gold (Tet^r^ Δ (*mcrA*)*183* Δ (*mcrCB-hsdSMR-mrr*)*173 endA1 supE44 thi-1 recA1 gyrA96 relA1 lac* Hte [F´ *proAB lacI*^q^*Z*Δ*M15* Tn*10* (Tet^r^) Amy Cam^r^]; Agilent Technologies, Santa Clara, CA, USA) was used as a host for plasmids.

**Table 1:** Strains used in this study.

Strains	Genotype	Source
H207	*MAT***a** *his3*-Δ*1 leu2*-*3*,*112 trp1*-*289 ura3*-*52 sst1*-*2*	[4]
H317	*cch1*Δ::*HIS3* in H207	[22]
H321	*cnb1*Δ::*HIS3* in H207	[36]
H701	*ecm7*Δ::HIS3* in H207	This study
H721	*ecm7*Δ::*HIS3 cnb1*Δ::*HIS3* in H207	This study
H717	*cch1*Δ::*HIS3 ecm7*Δ::*HIS3* in H207	This study
SK201	*scs2*Δ::*TRP1* in H207	This study
SH701	*scs2*Δ::*TRP1 ecm7*Δ::*HIS3* in H207	This study
SK3011	*scs2*Δ::*kanMx4 cch1*Δ::*HIS3* in H207	This study
NMY32	*MAT***a** *his3*Δ*200 trp1-901 leu2-3*,*112 ade2 LYS2*::(*lexA*op)_4_-*HIS3 URA3*::(*lexA*op)_8_-*lacZ ade2*::(*lexA*op)_8_-*ADE2 GAL4*	Dualsystems Biotech AG (Zürich, Switzerland)

*A dubious open reading frame (ORF) named *YLR444C* (303 bp) was identified that widely overlapped with the 3'-region of the *ECM7* ORF (1,347 bp) in the opposite direction. When we made the *ecm7*Δ::*HIS3* mutant, the *YLR444C* ORF was also deleted. Therefore, the resulting genotype was expected to be *ecm7*Δ-*ylr444c*Δ::*HIS3*. However, we confirmed that the *ylr444c*Δ::*URA3* single mutant had no phenotype regarding cell viability or Ca^2+^ accumulation under our experimental conditions. Therefore, we did not describe the dubious *ylr444c*Δ mutation in this study.

**Table 2:** Plasmids used in this study.

Plasmids	Characteristics	Source
YCplac111	*LEU2 ARS1 CEN4 amp*^r^	[37]
YCpE-ECM7	P_*ECM7*_*-ECM7-*T_*ADH1*_ *LEU2 ARS1 CEN4 amp*^r^	This study
YCpE-ECM7^1-322^	P_*ECM7*_*-ECM7*^*1-322*^*-*T_*ADH1*_ *LEU2 ARS1 CEN4 amp*^r^	This study
YCpE-ECM7^1-412^	P_*ECM7*_*-ECM7*^*1-412*^*-*T_*ADH1*_ *LEU2 ARS1 CEN4 amp*^r^	This study
YCpE-ECM7^Δ429–432^	P_*ECM7*_*-ECM7*^*Δ429-432*^*-*T_*ADH1*_ *LEU2 ARS1 CEN4 amp*^r^	This study
YCpE-ECM7^1-432^	P_*ECM7*_*-ECM7*^*1-432*^*-*T_*ADH1*_ *LEU2 ARS1 CEN4 amp*^r^	This study
pBC111	*LEU2 ARS1 CEN4 amp*^r^ ColE1-*ori*-*rop*	[22]
pBCT-CCH1H	P_*TDH3*_-*CCH1H-*T_*ADH1*_ *LEU2 ARS1 CEN4 amp*^r^ ColE1-*ori*-*rop*	[22]
pBCT-CCH1H-EGFP	P_*TDH3*_-*CCH1H*-*EGFP*-T_*ADH1*_ *LEU2 ARS1 CEN4 amp*^r^ ColE1-*ori*-*rop*	[22]
pBCY-BTC	P_*CYC1*_-*Cub-LexA-VP16*-T_*CYC1*_ *LEU2 ARS1 CEN4 amp*^r^ ColE1-*ori-rop*	This study
pBCY-BTC-CCH1H	P_*CYC1*_-*CCH1H*-*Cub-LexA-VP16-* T_*CYC1*_ *LEU2 ARS1 CEN4 amp*^r^ ColE1-*ori-rop*	This study
YCpT-NubG-FL5N	P_*TDH3*_-*NubG*-*FL5*-T_*ADH1*_ *URA3 ARS1 CEN4 amp*^r^	This study
YCpT-NubG-FL5-SCS2	P_*TDH3*_-*NubG*-*FL5*-*SCS2*-T_*ADH1*_ *URA3 ARS1 CEN4 amp*^r^	This study

P_*TDH3*_, *TDH3* promoter; P_*ECM7*_, *ECM7* promoter; T_*ADH1*_, *ADH1* terminator; P_*CYC1*_, *CYC1* promoter; T_*CYC1*_, *CYC1* terminator.

### Media

Synthetic media SD.Ca100 and SD were prepared as described previously [4]. SD.Ca100 contained 100 μM CaCl_2,_ while SD medium contained 681 μM CaCl_2_. YPD medium contained 1% Bacto-yeast extract, 2% Bacto-peptone, and 2% glucose.

### Ca^2+^ accumulation assay

The accumulation of Ca^2+^ was measured according to a previously described method [4]. Briefly, cells grown in SD.Ca100 medium at 30˚C to the exponential phase were incubated for 2 h with ^45^CaCl_2_ (185 kBq/ml; 1.85 kBq/nmol; PerkinElmer, Waltham, MA, USA) and 6 μM α-factor. Aliquots (duplicate) of the cultures were collected, filtered through Millipore filters (type HA; 0.45 μm, Merck, Tokyo, Japan) that had been presoaked in 5 mM CaCl_2_, and washed five times with the same solution. The radioactivity retained on the filters was counted with the scintillation cocktail, Emulsifier-Safe (PerkinElmer) in a liquid scintillation counter (Beckman Coulter LS 6500, Tokyo, Japan).

### Viability assay

Cells were grown in SD.Ca100 medium and exposed to 6 μM α-factor as described above. After an 8-h exposure, the viability of cells was assessed according to the methylene blue method described by Iida *et al*. [21]. This method is based on the ability of viable cells to reduce methylene blue to the colorless leukomethylene blue. Nonviable cells cannot reduce the dye and stain blue. Therefore, methylene blue-negative cells are viable and methylene blue-positive cells are nonviable. Viability was expressed as the percentage of the number of methylene blue-negative cells in the total number of cells. The number of methylene blue-negative cells is comparable to colony-forming units [21].

### Western blotting

Western blotting was performed according to a previously described method [4]. To determine the content of Cch1 in cells lacking *ECM7* and/or *CNB1*, crude extracts were subjected to SDS-PAGE followed by Western blotting. For co-immunoprecipitation experiments, proteins immunoprecipitated with an anti-Cch1 antibody was analyzed by SDS-PAGE and Western blotting. The antibodies used were a rabbit polyclonal anti-Cch1 antibody [22], mouse monoclonal anti-FLAG M2 antibody (Sigma-Aldrich Japan, Cat. No. F3165, Tokyo, Japan), mouse monoclonal anti-Pma1 antibody (GenScript Japan, Cat. No. A00101, Tokyo, Japan) and a rabbit polyclonal anti-enolase [23].

### Fluorescence microscopy

The subcellular localization of Cch1-EGFP in cells treated for 2 h with 6 μM α-factor was visualized using a confocal laser scanning microscope (LSM 780, Carl Zeiss Microscopy, Shinjuku, Tokyo, Japan) using excitation wavelengths of 488 nm.

### Co-immunoprecipitation

Cells (total of ~1 x 10^8^ cells) growing in SD medium were harvested, washed once with Milli-Q water, resuspended in BB-buffer (10 mM Tris-HCl [pH 7.5], 300 mM sorbitol, 100 mM NaCl, 5 mM MgCl_2_, 1 mM PMSF, and 1 tablet/10 ml of protease inhibitors [Roche Diagnostics, Basel, Switzerland]), and then lysed with glass beads by vortexing. Crude extracts were ultracentrifuged at 55,000 rpm (137,000 x *g*) at 4˚C for 30 min. The pellet (membrane fraction) was washed once with BB-buffer by ultracentrifugation and resuspended in 1 ml of IP-buffer (50 mM Tris-HCl [pH 8.0], 1.0% Triton X-100, 150 mM NaCl, 2 mM EDTA, 1 mM PMSF, and 1 tablet/10 ml of protease inhibitors) and left to stand at 4˚C for 60 min to be solubilized. The solution was ultracentrifuged at 55,000 rpm at 4˚C for 30 min and the supernatant was mixed and incubated with 30 μl of nProtein A Sepharose 4 Fast Flow (GE Healthcare, CT, USA) at 4˚C for 60 min with rotation. The mixture was centrifuged at 12,000 rpm for 1 min and the supernatant was mixed with rabbit anti-Cch1 (3.4 μl; 2.5 μg) and incubated at 4˚C for 60 min with rotation. nProtein A Sepharose 4 Fast Flow (50 μl) was supplemented to the mixture, which was incubated again at 4˚C for 60 min with rotation. The mixture was centrifuged at 12,000 rpm for 1 min, and the pellet was washed twice with centrifugation in IP-buffer and then washed once with wash-buffer (50 mM Tris-HCl [pH 8.0]). The final pellet was dissolved in SDS-sample buffer (50 mM Tris-HCl [pH 6.8], 4% SDS, 10% glycerol, 0.05% bromophenol blue, and 5% ß-mercaptoethanol). After heating at 95˚C for 5 min followed by centrifugation at 15,000 rpm at 25˚C, the supernatant was subjected to SDS-PAGE and a Western blot analysis or stocked at -20˚C until used. Western blots were used to detect Cch1, FLAG-tagged Scs2, and Pma1.

### Split-ubiquitin yeast two-hybrid screening

The yeast screening strain NMY32 (*MAT***a** *his3*Δ*200 trp1-901 leu2-3*,*112 ade2 LYS2*::(*lexA*op)_4_-*HIS3 URA3*::(*lexA*op)_8_-*lacZ ade2*::(*lexA*op)_8_-*ADE2 GAL4*) carrying the plasmid pBCY-BTC-CCH1H, which expresses the Cch1-Cub-LexA-VP16 fusion protein as bait, was transformed with the *S*. *cerevisiae* cDNA library/NubG-x (Dualsystems Biotech AG, Zürich, Switzerland), which was designed to express prey proteins, and incubated on SD plates + Ade—(His, Leu, and Trp) at 30˚C. The transformants obtained were streaked on new plates with the same nutrient composition and incubated for several days. In the second screening, transformants were grown for 2–3 days on SD plates—(Ade, His, Leu, and Trp). cDNA sequences on prey plasmids were then elucidated.

### Statistical analysis

The significance of differences was evaluated using an unpaired Student’s *t*-test, with a maximum *p* value of < 0.05 being required for significance.

## Results

### Ecm7 is required for HACS in calcineurin-deficient cells

**Fig 2:**
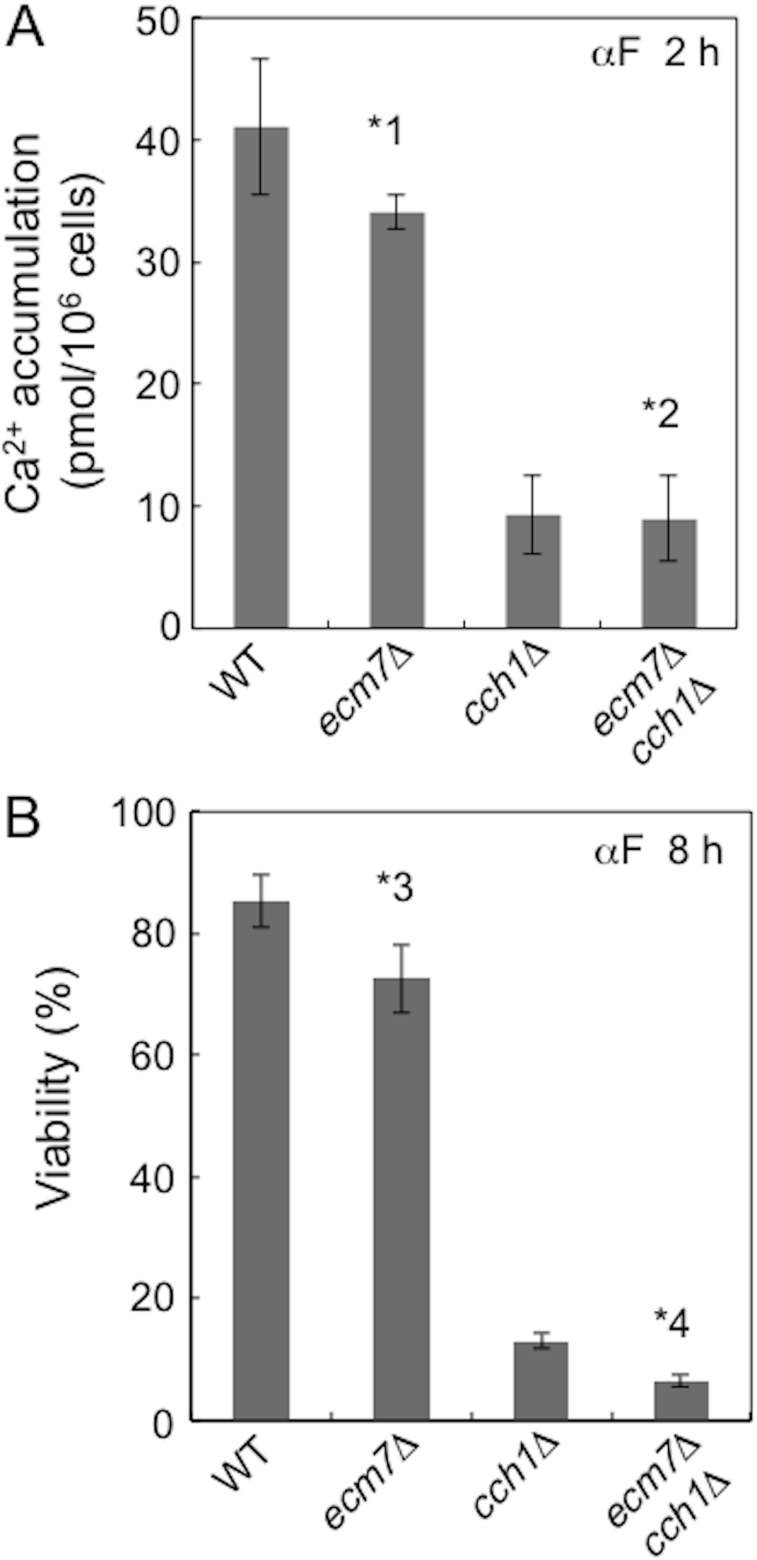
The deletion of *ECM7* results in a slight decrease in Ca^2+^ accumulation and viability in *CNB1*^+^ cells. Cells grown in SD.Ca100 medium were treated with 6 μM α-factor and incubated. Ca^2+^ accumulation and the viability of cells were assessed 2 h and 8 h after the addition of α-factor. *1, *p* < 0.05 (WT *vs*. *ecm7*Δ); *2, *p* > 0.05 (*cch1*Δ *vs*. *cch1*Δ *ecm7*Δ); *3, *p* < 0.05 (WT *vs*. *ecm7*Δ); *4, *p* < 0.05 (*cch1*Δ *vs*. *cch1*Δ *ecm7*Δ). Data are the mean ± SD of three independent experiments.

In order to study the role of Ecm7, we employed low Ca^2+^ medium containing 100 μM CaCl_2_, named SD.Ca100, in which HACS becomes active in α-factor-treated cells [4,7]. We incubated the *ecm7*Δ mutant as well as its control, the *cch1*Δ mutant and their parental *ECM7*^+^ *CCH1*^+^ strain (designated here as the wild type) with α-factor for 2 h for Ca^2+^ accumulation assays and 8 h for viability assays. The results obtained showed that only slightly less Ca^2+^ accumulated in the *ecm7*Δ mutant (by 17%) than in the wild-type strain, while Ca^2+^ accumulation was reduced by 77% in the *cch1*Δ mutant (Fig 2A). The *ecm7*Δ *cch1*Δ double mutant quantitatively showed the same accumulation of Ca^2+^ as the *cch1*Δ mutant. Viability assays showed similar results between the *ecm7*Δ and *cch1*Δ mutants and wild-type strain (Fig 2B). These results suggest that Ecm7 only negligibly contributes to HACS in cells exposed to α-factor, even in low Ca^2+^ medium.

**Fig 3:**
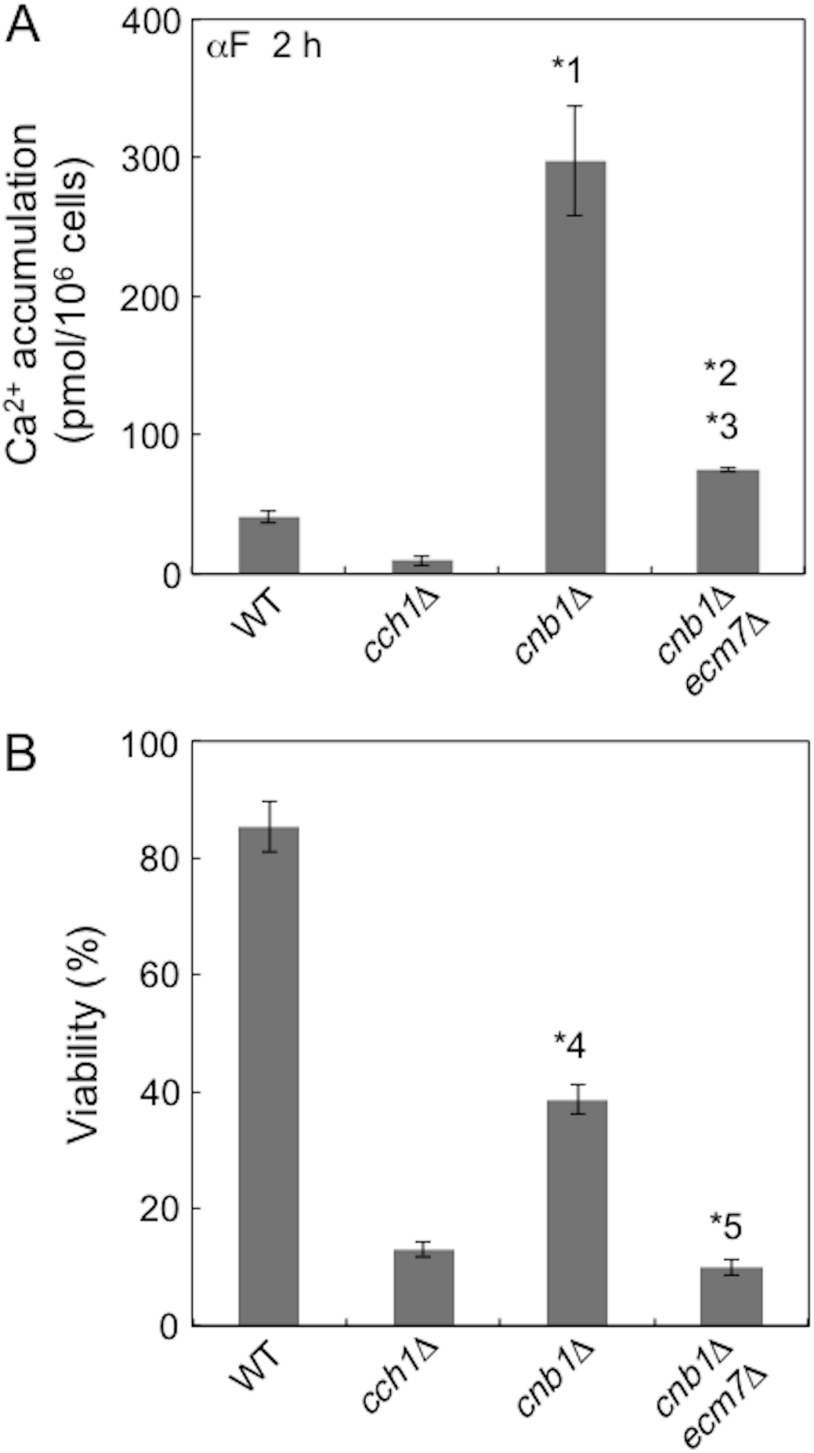
The deletion of *ECM7* results in a marked decrease in Ca^2+^ accumulation and viability in *cnb1*Δ cells. Experimental conditions and procedures were the same as those described in the legend to Fig 2. *1, *p* < 0.05 (WT *vs*. *cnb1*Δ); *2, *p* < 0.05 (*cnb1*Δ *vs*. *cnb1*Δ *ecm7*Δ); *3, *p* < 0.05 (WT *vs*. *cnb1*Δ *ecm7*Δ); *4, *p* < 0.05 (WT *vs*. *cnb1*Δ); *5, *p* < 0.05 (*cch1*Δ *vs*. *cnb1*Δ *ecm7*Δ). Data are the mean ± SD of three independent experiments.

We then examined the contribution of Ecm7 to HACS using the *cnb1*Δ mutation, which produces no calcineurin activity, because a previous study reported that α-factor-induced Ca^2+^ accumulation in the *ecm7*Δ mutant was approximately 50% less than that in the wild-type strain, even in YPD medium, when the calcineurin inhibitor, FK506 was added [8]. Our results showed that, in SD.Ca100 medium, α-factor-induced Ca^2+^ accumulation was 7.4-fold higher in the *cnb1*Δ mutant than in the wild-type strain and also that the high level of accumulation in the *cnb1*Δ mutant was markedly decreased to one-fourth when *ECM7* was deleted (Fig 3A). Viability assays showed that the viability of the *cnb1*Δ mutant was further lowered in the *ecm7*Δ *cnb1*Δ mutant (Fig 3B). Since HACS or its regulator is not dephosphorylated by calcineurin in the *cnb1*Δ mutant, the above results suggest that Ecm7 is selectively required for a phosphorylated form of HACS or its regulator. Alternatively, it is possible that a phosphorylated form of Ecm7 is important for the activity of HACS or its regulator.

**Fig 4:**
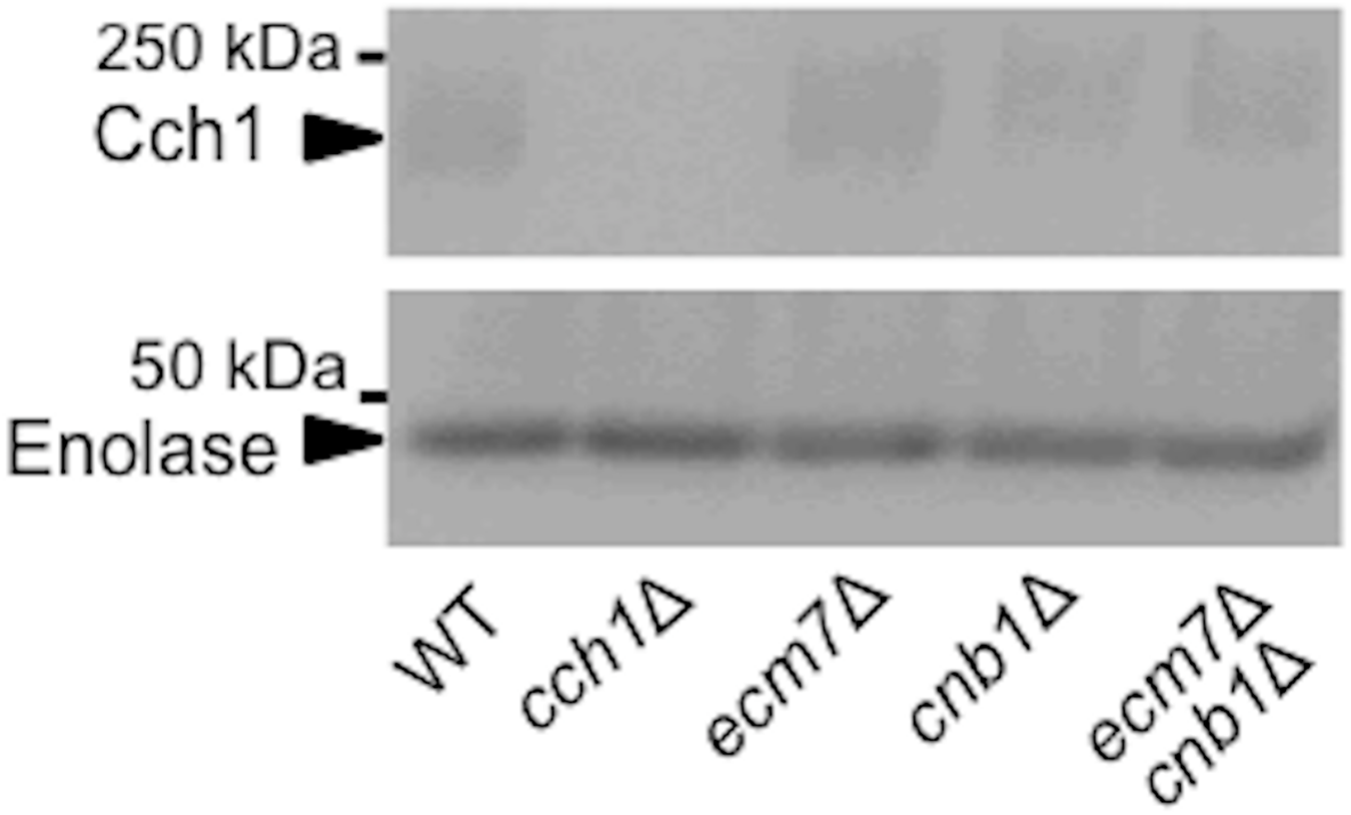
The deletion of *ECM7* and *CNB1* does not affect the amount of Cch1. Cells with various deletion mutations were exposed for 2 h to 6 μM α-factor and crude extracts were prepared. The amount of Cch1 in these cells was assessed by Western blotting. Enolase is an internal marker.

In the Western blot analysis and confocal laser scanning microscopy, we found that the deletion of *ECM7* and/or *CNB1* did not affect the content of the Cch1 protein (Fig 4) or the subcellular localization of Cch1-EGFP expressed from a strong *TDH3* promoter (S1 Fig). It should be noted that expression from a strong promoter could sometimes lead to mislocalization of a protein of interest. In fact, while the localization of Cch1-Myc expressed from the *CCH1* promoter is reported to be localized exclusively in the plasma membrane [24], Cch1-EGFP expressed from the *TDH3* promoter was localized in not only the plasma membrane but also the ER membrane (S1 Fig). Nevertheless, we would like to point out that the fluorescence images of Cch1-EGFP were very similar between *cnb1*Δ, *ecm7*Δ *cnb1*Δ and wild-type cells.

### The cytoplasmic C-terminal region of Ecm7 is partially necessary

The cytoplasmic C-termini of human γ subunit isoforms are highly divergent, except for the terminal seven amino acid residues, which are conserved [25]. Similarly, the amino acid alignment of Ecm7 and its orthologs from several species of the genus *Saccharomyces* revealed that regions near the cytoplasmic C-termini were divergent, whereas the terminal 37 amino acid residues were well conserved. In addition, mass spectrometry-based proteomics identified phosphorylation sites at seven Ser residues in the C-terminal region ranging from Ser^360^ to the C terminus (Phe^448^) [26] (Fig 1).

**Fig 5:**
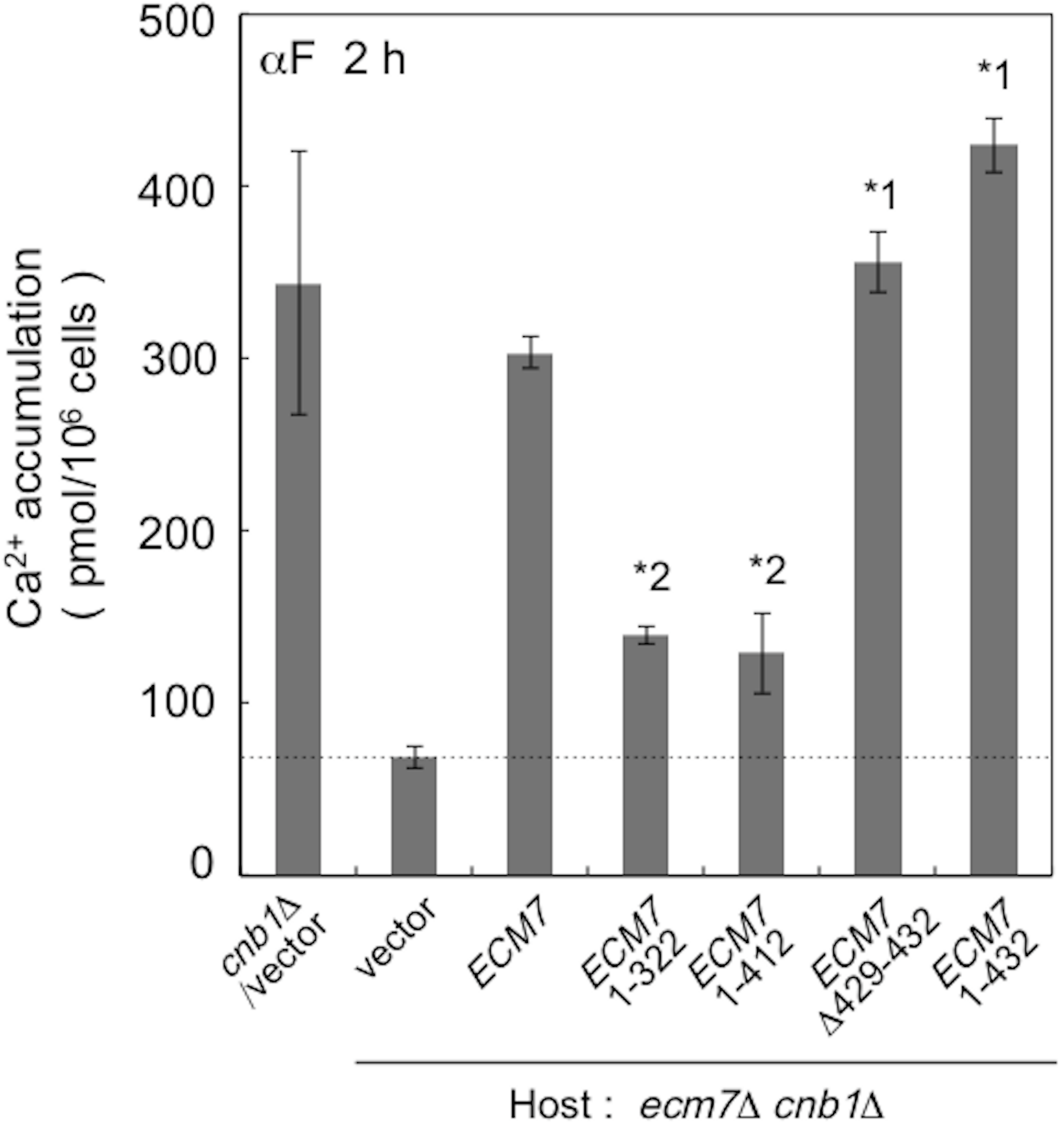
C-terminal truncations after amino acid residues 322 and 412 decrease Ecm7 activity. Experimental conditions and procedures were the same as those described in the legend to Fig 2. The positions of a deletion (Δ429–432) and small truncation (1–432) were also described under the graph. *1, *p* > 0.05 (*cnb1*Δ*/*vector *vs*. *ecm7*Δ *cnb1*Δ*/*mutated *ECM7s*); *2, *p* < 0.05 (*cnb1*Δ*/*vector *vs*. *ecm7*Δ *cnb1*Δ*/*mutated *ECM7s*). Data are the mean ± SD of three independent experiments.

In order to examine the importance of these regions and some of the phosphorylation sites, we performed truncation analysis. The C-terminally truncated forms of Ecm7, Ecm7^1-322^ lacking all of the seven phosphorylation sites and Ecm7^1-412^ lacking three C-terminal phosphorylation sites (Fig 1), were expressed in the *ecm7*Δ *cnb1*Δ mutant and Ca^2+^ accumulation was measured (Fig 5). After the subtraction of background Ca^2+^ accumulation in cells carrying an empty vector from that in cells expressing each mutant Ecm7 (see the dotted line in Fig 5), we found that the Ca^2+^ accumulation activities of Ecm7^1-322^ and Ecm7^1-412^ were significantly reduced, but were still maintained at approximately one-fourth that of the wild-type Ecm7 (Fig 5). This result indicates that the C-terminally truncated regions, which contain the seven phosphorylation sites, are required for the full activity of Ecm7, but are not necessarily essential. In order to examine the validity of this suggestion, we constructed the following three mutant proteins: (1) Ecm7^ΔS429-S432^, a mutant Ecm7 protein with the deletion of the region (Ser^429^ to Ser^432^) containing the two phosphorylation sites and (2) Ecm7^1-432^, a mutant Ecm7 protein with the deletion of the C-terminal region ranging from Glu^433^ to the C terminus. We then measured their Ca^2+^ accumulation activities. The results obtained showed that the two mutant Ecm7 proteins exhibited normal Ca^2+^ accumulation activities. Since Ecm7^1-412^ showed reduced activity, the region between 413 and 428 amino acid residues appears to be important for the activity of Ecm7, although its contribution is restrictive.

### Scs2 positively regulates HACS

**Fig 6:**
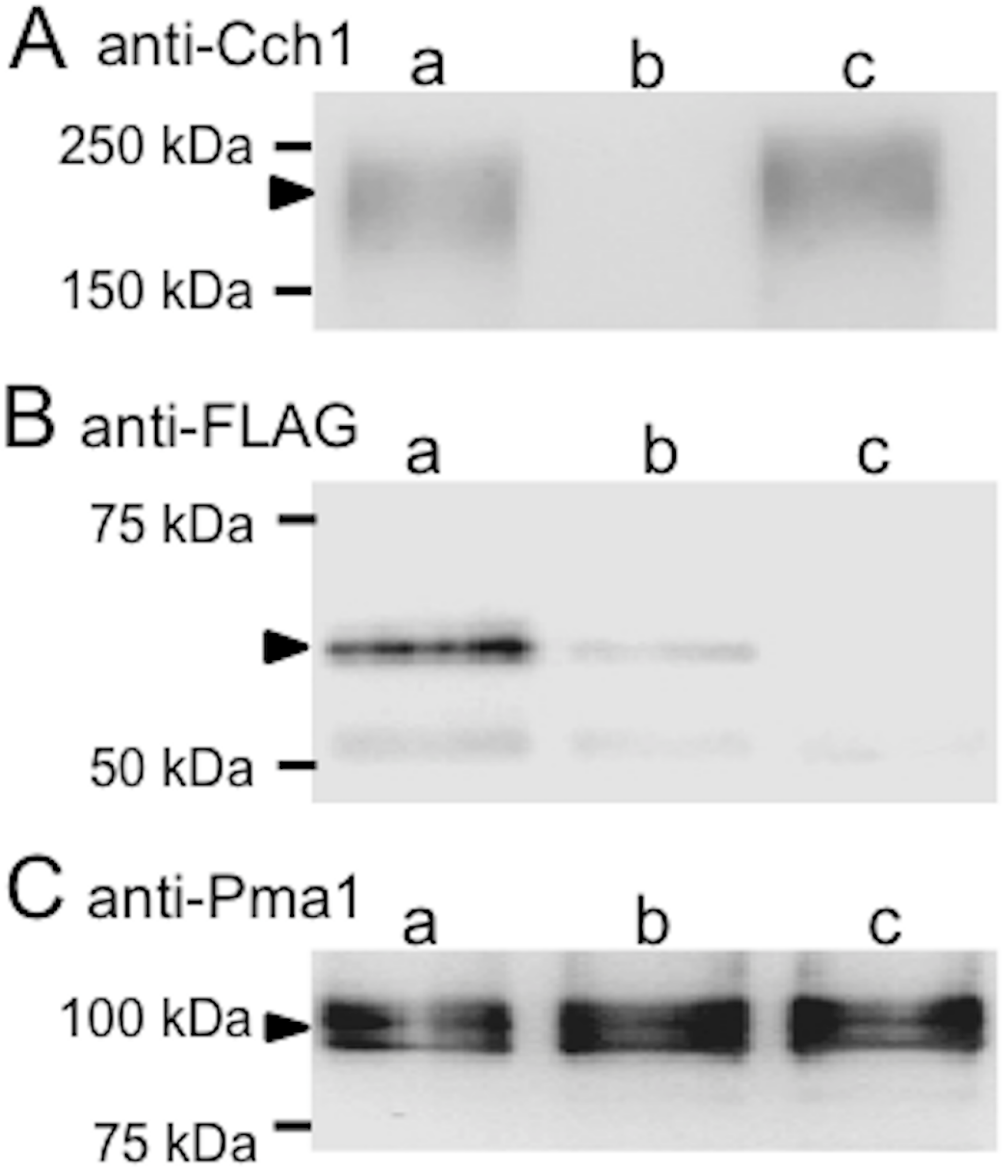
Co-immunoprecipitation and co-localization. (A, B) The membrane fraction was immunoprecipitated with an anti-Cch1 antibody and co-immunoprecipitated proteins were subjected to Western blotting in order to detect FLAG-tagged Scs2. (C) An aliquot of the membrane fraction used above was directly subjected to Western blotting to detect the plasma membrane marker protein Pma1. The strain used was SK3011 (*scs2*Δ *cch1*Δ) carrying either pBCT-CCH1H + YCpT-NubG-FL5-SCS2 (*lane a*), pBC111 (empty vector) + YCpT-NubG-FL5-SCS2 (*lane b*), or pBCT-CCH1H + YCpT-NubG-FL5N (empty vector) (*lane c*). Arrowheads point to the Cch1 (A), FLAG-tagged SCS2 (B) and Pma1 (C) proteins. In *lane b*, a faint band was observed for an unknown reason.

In the search for membrane proteins that potentially interact with HACS, we screened candidates with the split-ubiquitin membrane-based yeast two-hybrid system [27, 28], using Cch1 as bait and *S*. *cerevisiae* cDNA libraries as prey. We obtained 12 candidates, among which Scs2 was the most frequently detected. Details on this screening will be published elsewhere; we herein focused on Scs2, particularly its genetic study in terms of HACS regulation because this protein had been reported as a member of LACS [8]. We confirmed that N-terminally tagged FLAG-Scs2 co-immunoprecipitated with Cch1 when anti-Cch1 antibodies were used as the primary antibody followed by Western blotting with the anti-FLAG antibody (Fig 6A–6C).

**Fig 7:**
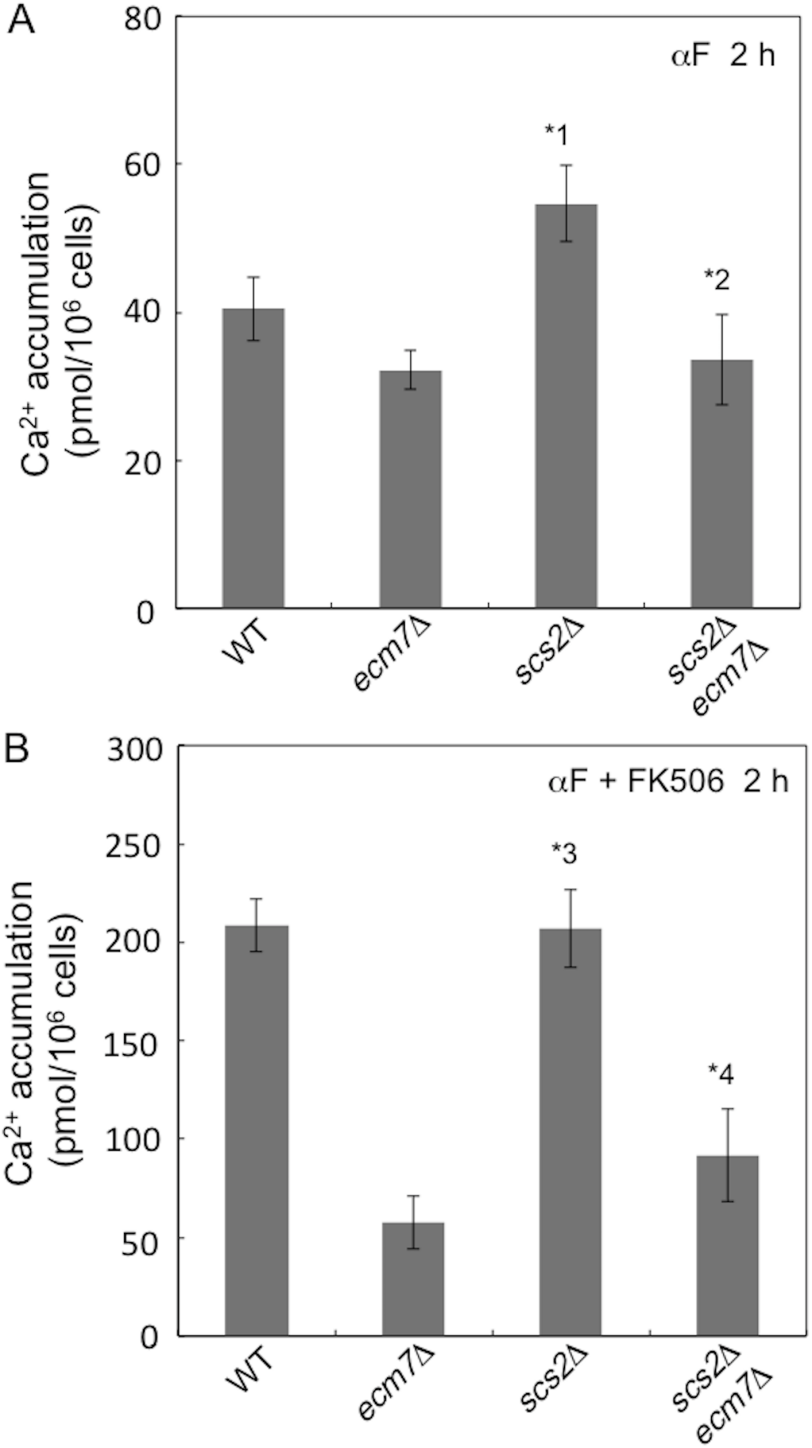
The deletion of *SCS2* increases Ca^*2+*^ accumulation in calcineurin-active cells, but not in calcineurin-inactivated cells. Experimental conditions and procedures were the same as those described in the legend to Fig 2. Cells were incubated for 2 h with 6 μM α-factor only (A) or with 6 μM α-factor and 2.0 μg/ml FK506 (B). *1, *p* < 0.05 (*scs2*Δ *vs*. WT); *2, *p* > 0.05 (*scs2*Δ *ecm7*Δ *vs*. *ecm7*Δ); *3, *p* > 0.05 (*scs2*Δ *vs*. WT); *4, *p* < 0.05 (*scs2*Δ *ecm7*Δ *vs*. *ecm7*Δ). Data are the mean ± SD of three independent experiments.

Scs2 is a type II integral membrane protein with a single C-terminal transmembrane segment that localizes at the ER membrane, and is a homolog of VAP [15]. In order to investigate the role of Scs2 in Ca^2+^ influx through HACS, the *scs2*Δ mutant was constructed and examined for Ca^2+^ accumulation 2 h after exposure to α-factor in SD.Ca100 medium. The results obtained showed that 35% more Ca^2+^ accumulated in the *scs2*Δ mutant than in the wild-type strain (Fig 7A). This enhancement was not observed in the *scs2*Δ *ecm7*Δ mutant, suggesting that Scs2 negatively regulates HACS activity and that Ecm7 has a positive effect on the function of a system regulated negatively by Scs2 although the molecular basis of this effect is unknown. In contrast, when FK506 was added to the medium, Ca^2+^ accumulation was not enhanced in the *scs2*Δ mutant (Fig 7B). Therefore, the role of Scs2 in the regulation of HACS activity appears to potentially depend on the phosphorylation status of HACS or its regulator.

*SCS2* has a paralog, *SCS22* [29]. The *scs22*Δ mutant showed the wild-type levels of Ca^2+^ accumulation and cell viability after exposure to α-factor and the *scs2*Δ *scs22*Δ double mutant showed the same phenotypes as the *scs2*Δ mutant (data not shown), suggesting that Scs22 is not involved in the regulation of the Cch1/Mid1 channel under our assay conditions.

## Discussion

In the present study, we identified two regulators of HACS, Ecm7 and Scs2, under the experimental conditions that activate HACS, *i*.*e*. under low extracellular Ca^2+^ conditions: the former is a positive regulator, while the latter is a negative regulator. We showed that Ecm7, a yeast homolog of animal VGCC γ subunits, was hardly necessary for α-factor-induced HACS activity when calcineurin was functional in cells incubated in low Ca^2+^ medium. However, when the *CNB1* gene encoding a subunit of this protein phosphatase was deleted or this enzyme was inhibited by FK506, Ecm7 switched to a strong positive regulator. Although Ecm7 possesses seven phosphorylation sites in the intracellular C-terminal region [26], these were suggested to be nonessential for the function of Ecm7 (Fig 5; see the next paragraph). Thus, a phosphorylated form of Ecm7 would not be required for HACS activation in calcineurin-deficient cells. Therefore, Ecm7 appears to selectively activate the phosphorylated form of HACS or its regulator. One of the Ecm7 targets may be Cch1 because the entire molecule of Mid1 is localized extracellularly and, thus, cannot be dephosphorylated by cytoplasmic calcineurin (Iida K *et al*., manuscript in preparation). The calcineurin deficiency-dependent activation of HACS was also reported for *S*. *cerevisiae* cells exposed to tunicamycin, an ER stressor; however, the involvement of Ecm7 was negligible, and this may have been because YPD medium, which is rich in nutrients and Ca^2+^, was used to cultivate cells [8]. A previous study demonstrated that HACS hardly functions in this rich medium [7].

A truncation analysis showed that the large cytoplasmic C-terminal region of Ecm7 was partially responsible for the full Ca^2+^ influx activity of HACS, suggesting that the seven phosphorylation sites present in this region are less important. Particularly, two C-terminally truncated forms, Ecm7^1-322^ and Ecm7^1-412^, had the same activity each other (Fig 5), suggesting that the amino acid residue 323–412 segment containing four phosphorylation sites (Ser^360^, Ser^381^, Ser^387^, and Ser^394^) is not involved in Ecm7 function. In addition, Ecm7^Δ429–432^ lacking two phosphorylation sites (Ser^429^ and Ser^432^) and Ecm7^1-432^ lacking the phosphorylation site Ser^435^ did not lose the activity at all (Fig 5). Therefore, it is suggested that all of the seven phosphorylation sites do not contribute to the full activity of Ecm7 at least under the experimental conditions we employed. Among the eight members of mammalian γ subunits (γ_1_ to γ_8_), only one member, γ_2_, was found to be phosphorylated in the cytoplasmic C-terminal region [30, 31]. Since Ecm7 has four transmembrane segments that are highly conserved among the VGCC γ subunit family, the four transmembrane segments with a large extracellular loop between segments 1 and 2 may play a positive regulatory role.

Another regulator, Scs2, was newly discovered by the screening of Cch1-interacting membrane proteins with the split-ubiquitin membrane-based yeast two-hybrid system. The deletion of the *SCS2* gene enhanced α-factor-induced HACS activity by 35% and this enhancement was canceled in the absence of Ecm7 or when cells were incubated with the calcineurin inhibitor FK506. Since Scs2 is shown to be phosphorylated at Ser^106^, Ser^153^, Thr^200^, Ser ^201^, Ser^203^, and Thr^204^ [26, 32], it is possible that HACS activity is dually regulated by Ecm7 and Scs2 in a calcineurin-dependent manner. The following hypothesis could be possible: when calcineurin is non-functional due to a low cytosolic Ca^2+^ concentration, Ecm7 may activate a phosphorylated form of HACS or its regulator. However, this activation may not be applied to its dephosphorylated form. When calcineurin is functional due to a high cytosolic Ca^2+^ concentration, Scs2 may repress the activity of a dephosphorylated form of HACS or its regulator, but may not affect its phosphorylated form. This hypothesis warrants further studies looking at HACS phosphorylation and dephosphorylation.

Scs2 is localized to the cortical ER membrane, at which it acts as an ER-to-plasma membrane-tethering protein [18]. Cch1 is located in the plasma membrane [24], while Mid1 is localized to the ER and plasma membranes [33–36]. Since the Cch1/Mid1 channel or HACS is activated by ER stress [9], it is tempting to speculate that Scs2 transduces an ER signal to the Cch1/Mid1 channel in order to regulate Ca^2+^ influx through the plasma membrane.

In conclusion, we demonstrated that the VGCC γ subunit homolog Ecm7 functioned as a strong positive regulator of Cch1 in calcineurin-deficient *cnb1*Δ cells exposed to α-factor in low-Ca^2+^ medium. However, this positive regulation is weak in *CNB1*^+^ cells. We identified the cortical ER resident protein Scs2 as a novel interactor of Cch1 and demonstrated that Scs2 negatively regulated Cch1 in calcineurin-functional cells.

## Supporting information

S1 Fig The deletion of *ECM7* and *CNB1* does not affect the subcellular localization of Cch1-EGFP. Cch1-EGFP images of cells treated for 2 h with 6 μM α-factor were obtained by confocal scanning fluorescence microscopy. Cch1-EGFP was expressed from the *TDH3* promoter and appeared to be localized in the ER and plasma membranes in the *cnb1*Δ and *ecm7*Δ *cnb1*Δ mutants as well as in the wild-type strain.
(TIFF)
