# Supplementary figures and images for: Genetic analysis of the regulation of the voltage-gated calcium channel homolog Cch1 by the γ subunit homolog Ecm7 and cortical ER protein Scs2 in yeast
Source: PLoS One. 2017 Jul 24;12(7):e0181436. doi: 10.1371/journal.pone.0181436 (PMC5524387; doi:10.1371/journal.pone.0181436)

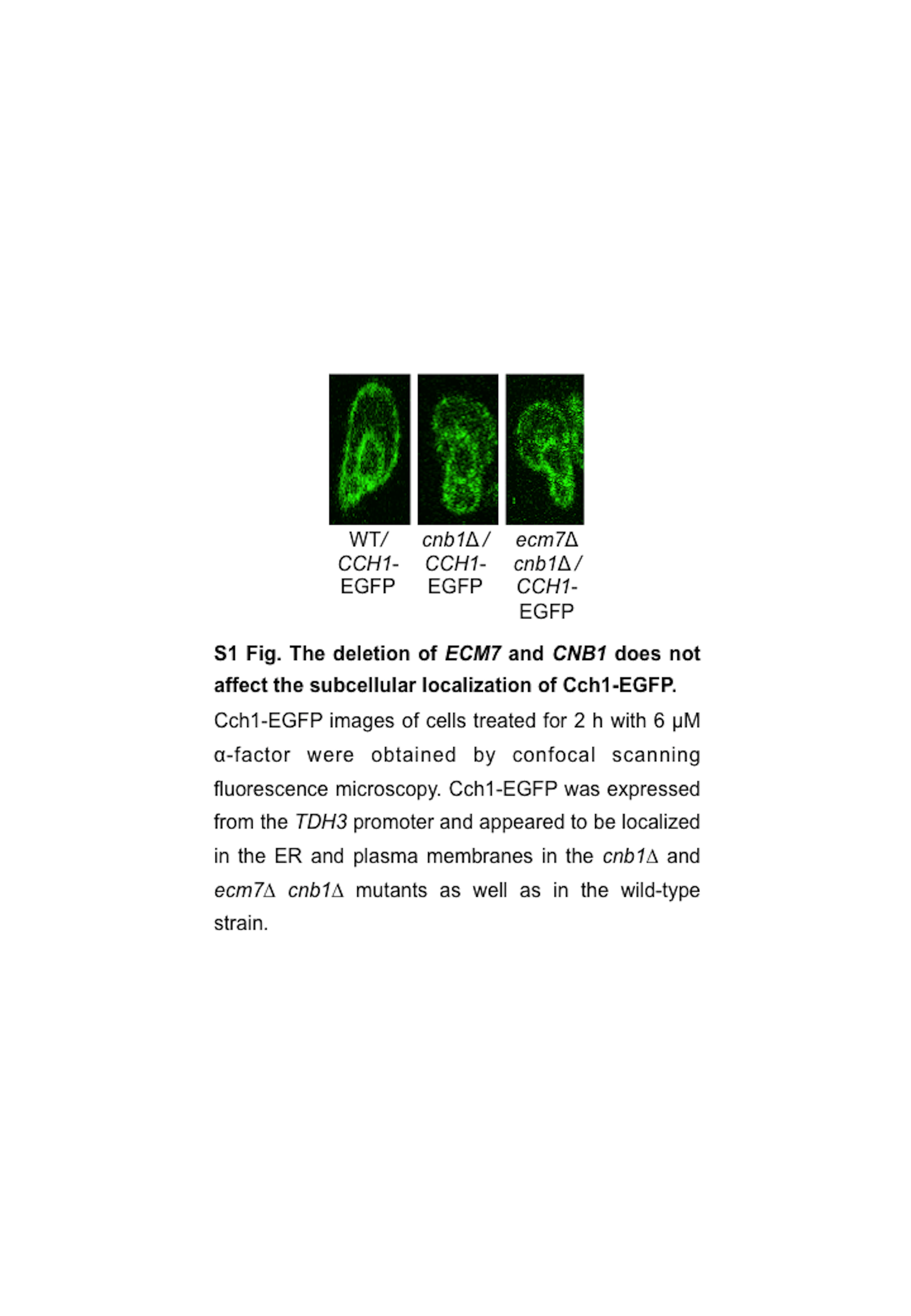

Supplement: S1 Fig — Cch1-EGFP images of cells treated for 2 h with 6 μM α-factor were obtained by confocal scanning fluorescence microscopy. Cch1-EGFP was expressed from the TDH3 promoter and appeared to be localized in the ER and plasma membranes in the cnb1Δ and ecm7Δ cnb1Δ mutants as well as in the wild-type strain. (TIFF) [file pone.0181436.s001.tiff]
